# “The Baltic Sea Germ”: A Case Report of Necrotizing Fasciitis following Vibrio vulnificus Infection

**DOI:** 10.1155/2022/5908666

**Published:** 2022-03-23

**Authors:** Heinz-Lothar Meyer, Christina Polan, Manuel Burggraf, Lars Podleska, Paula Beck, Hans-Ulrich Steinau, Marcel Dudda, Farhad Farzaliyev

**Affiliations:** ^1^Department for Trauma, Hand and Reconstructive Surgery, Essen University Hospital, Hufelandstraße 55 45147 Essen, Germany; ^2^Department for Tumor Orthopedics and Sarcoma Surgery, Essen University Hospital, Hufelandstraße 55 45147 Essen, Germany

## Abstract

Reported is an 80-year-old patient with septic shock from necrotizing fasciitis secondary to a Vibrio vulnificus (Vv) infection. The patient reports having been swimming in the Baltic Sea after a minor trauma to the left leg. Emergency superficial necrosectomy followed by intensive medical therapy was performed. Antibiotic therapy was initiated with a third-generation cephalosporin and a tetracycline. Vv was detected in the intraoperative microbiological smears. Instead of a leg amputation and a flap, due to the patient's age, a split skin covering to consolidate the wound was performed. Vv is a gram-negative rod bacterium of the genus Vibrio. Vv occurs in warm, low-salinity seawater (brackish water). In Germany, Vv occurs primarily in river mouths of the low-salinity Baltic Sea. Infections by Vv can occur through open wounds or by eating raw infected seafood, especially oysters. Infection via wounds often take a fulminant lethal course. Patients with chronic diseases, weakened immune system, and open wounds are particularly at risk. Infections with Vv are rare, but occur worldwide. Global warming is expected to spread Vv as water temperature increases and the dilution effect of sea level rise further decreases ocean salinity, and natural disasters promote the spread of Vv.

## 1. Introduction

Vibrio vulnificus (Vv) is a gram-negative rod-shaped bacterium of the genus Vibrio. Vv occurs naturally in warm and low-salt seawater or brackish water [1–3]. In Germany, Vv is found especially in river mouths of the low-salt Baltic Sea. A low salt concentration is ideal for growth, as found in the Baltic Sea [4]. Therefore, in studies of Baltic Sea estuaries, Vv was detected in significantly more samples than in samples from the North Sea [5]. Infections with Vv are rare but occur worldwide. Vv exists in an inactive state at cold water temperatures, such as those present in winter [6, 7]. If water temperature rises above 20°C in summer, Vv is activated. Once activated, the bacterium can maintain its activity for several weeks even when water temperatures drop [6, 7]. Vv infections can occur by swimming or wading in contaminated waters. The bacteria can enter through open wounds in the skin [2, 8]. A second route of transmission is the consumption of raw infected seafood, especially oysters [5, 9]. Symptoms following consumption of infected seafood include vomiting, diarrhea, abdominal pain, and blistering dermatitis [10]. The less common infection via wounds often takes a serious course to sepsis with a rapidly fulminant progression to septic shock [11]. Patients with chronic diseases, weakened immune system, and open wounds are particularly at risk. Older, male individuals with chronic liver disease, immunosuppression, or diabetes mellitus are the typical risk group for Vv infection and a fulminant course [6, 12]. Often, the affected extremities have to be amputated. The lethality of Vv infections is reported in the literature to approximately 25%. If the infection progresses to sepsis, the lethality rate increases to over 50%. Most deaths occur within the first 48 hours of infection [13]. There is no guideline treatment for Vv infection. A study from Taiwan demonstrated that the combined use of a third-generation cephalosporin with tetracyclines was associated with a better outcome [14, 15]. The most frequent reports come from the USA (East Coast), Japan, and Taiwan [13, 16]. In this case report, we present a fulminant Vv infection of the lower limb that was successfully treated without amputation, hence developing a highly differentiated squamous cell carcinoma of the affected limb in the long-term after split skin graft and chronic lymphedema four years after surviving the infection.

## 2. Case Presentation

**Figure 1:**
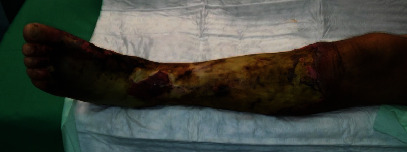
Necrotizing fasciitis of the left lower leg with Vv.

**Figure 2:**
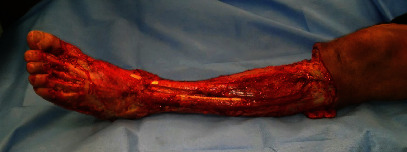
After emergency superficial necrosectomy of the necrotizing fasciitis of the left lower leg.

**Figure 3:**
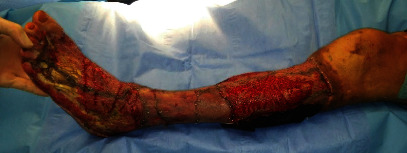
Left lower leg after the first split skin graft.

**Figure 4:**
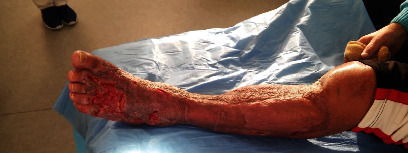
Left lower leg 3 months after split skin graft.

**Figure 5:**
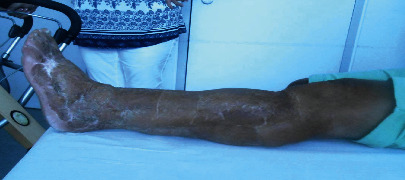
Two years after necrotizing fasciitis of the left lower leg with Vv.

**Figure 6:**
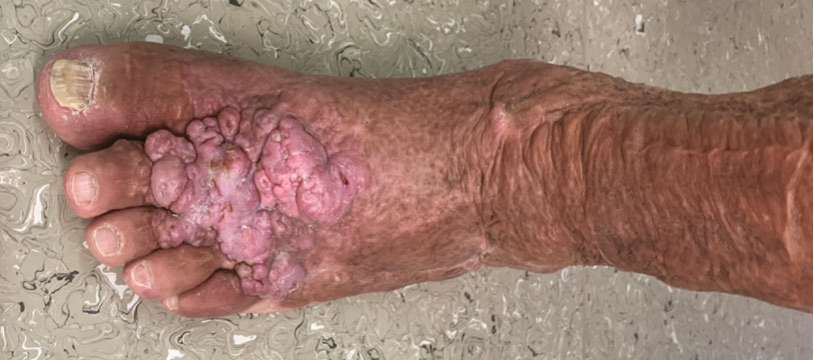
Highly differentiated squamous cell carcinoma of the skin on the dorsum of the left foot of the lower leg, four years after an infection by Vv.

**Figure 7:**
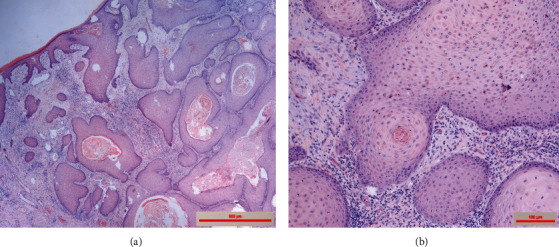
Histopathology Hematoxylin Eosin (HE) staining of the differentiated squamous cell carcinoma four years after surviving infection in the affected extremity.

Presented is a case of an 80-year-old patient who was on holiday on the German Baltic coast in September. He recalled a minor trauma to his left leg prior to swimming in the Baltic Sea several times. Afterwards, he noticed an infection in the area of the left lower leg, which rapidly developed into necrotizing fasciitis and septic shock. Initial treatment at the local hospital only consisted of superficial debridement. Subsequently, the patient was transferred to our hospital for further treatment. Due to the vital threat, an emergency necrosectomy was performed for necrotizing fasciitis of the left lower leg (Figures 1 and 2) followed by intensive care therapy. Antibiotic therapy was continued with Meronem and Clindamycin. Vv was detected in the microbiological swabs obtained intraoperatively. Instead of a leg amputation and a flap, due to the patient's age, a split skin covering to consolidate the wound was performed (Figures 3 and 4). Only areas in the dorsal region of the left foot and in the ventral region of the tibialis anterior tendon showed failed split skin grafts. Here, tangential ablation of the fibrin-covered tendons was performed at regular intervals. These areas granulated secondarily. After a two-month hospital stay, the patient was able to leave the clinic standing and walking safely (Figure 5). Four years after the initial vibrion infection, the patient exhibited an inconspicuous wound situation in the area of the skin graft region of the lower leg that had otherwise healed without irritation up to the ankles. The area of the forefoot at the metatarsophalangeal joints showed an approximately 7 × 4 cm large, smelly, and granulating wound surface on the extensor side. In the further course, hypergranulation tissue appeared on the dorsum of the left foot (Figure 6). Surgical resection of the hypergranulation tissue and covering of the defect with split skin of the left foot was performed. Histopathologic analysis of the intraoperative tissue samples ultimately resulted in the finding of the highly differentiated squamous cell carcinoma on the dorsum of the left foot of the affected limb after split skin graft and chronic lymphedema (Figures 6, 7(a), and 7(b)). The primarius was excised. In the further course, the patient could be released into the home environment after an early rehabilitation. A follow-up one year later showed another recurrence of the highly differentiated squamous cell carcinoma on the dorsum of the left foot which was excised again.

## 3. Discussion

The presented case shows a vital threatening Vv infection with an extremity-preserving therapy. The fulminant course in this patient shows the possible danger of Vv infections. Due to global warming, the water temperature of the oceans is increasing. The polar ice caps are melting, causing sea levels to rise and a dilution effect to reduce the salt concentration of the oceans [6]. The German Baltic coast is one of the most vulnerable areas due to low salinity and increasing warming. A review article by Baker-Austin et al. already discussed some increase in Vv cases in the Baltic Sea area as a result of climate change [17]. After the increasing number of natural disasters (floods, hurricanes, etc.), Vv can be detected in the flood plains [18]. As a result, infections caused by Vv can be expected to increase in the coming decades. Furthermore, Vv is the most common pathogen for infections with a lethal outcome associated with seafood consumption in the United States. There are increasing numbers in multidrug-resistant Vv to antibiotics in seafood. This has been attributed to the increase in fish and shellfish farms [9]. Since 1985, the annual numbers of Vv infections registered in Germany have also been steadily increasing. In particular, the above mentioned risk groups should be made more aware of the danger in the next years and medical doctors in the corresponding regions should be sensitized to it [8, 19]. This is especially true with regard to the affected regions in Germany where many health resorts are located, and thus, many people at risk are exposed to a possible Vv infection [20]. Regarding malignant degeneration in the long-term course, studies have already shown that squamous cell carcinomas have formed after split skin grafts. This applies to both the donor and the transplant site, regardless of the region of the body [21, 22]. The same has been observed in areas of the body with chronic lymphedema [23]. Hence, in the context of Vv infection, this association has not yet been adequately described or refuted in the literature. In the presented case, due to the extremity preserving procedure with large area split skin grafts after necrosectomy instead of amputation of the affected limb, the patient's initial quality of life certainly was significantly improved. Nevertheless, appropriate patient education seems at least highly recommendable.

## Data Availability

The patient presented has agreed to the anonymous presentation of his medical history.
